# Ultrashort time-to-echo T2* and T2* relaxometry for evaluation of lumbar disc degeneration: a comparative study

**DOI:** 10.1186/s12891-022-05481-9

**Published:** 2022-06-01

**Authors:** Li-Lan Wu, Li-Heng Liu, Sheng-Xiang Rao, Pu-Yeh Wu, Jian-Jun Zhou

**Affiliations:** 1 Department of Radiology, Xiamen Branch, Zhongshan Hospital, Fudan University, Xiamen, China; 2 Department of Radiology, Zhongshan Hospital, Fudan University, shanghai, China; 3 Shanghai Institute of Medical Imaging, shanghai, China; 4 GE Healthcare, Beijing, China

**Keywords:** Disc degeneration, Lumbar spine, Quantitative magnetic resonance imaging, T2*, UTE-T2*

## Abstract

**Background:**

To compare potential of ultrashort time-to-echo (UTE) T2* mapping and T2* values from T2*-weighted imaging for assessing lumbar intervertebral disc degeneration (IVDD),with Pfirrmann grading as a reference standard.

**Methods:**

UTE-T2* and T2* values of 366 lumbar discs (L1/2-L5/S1) in 76 subjects were measured in 3 segmented regions: anterior annulus fibrosus, nucleus pulposus (NP), and posterior annulus fibrosus. Lumbar intervertebral discs were divided into 3 categories based on 5-level Pfirrmann grading: normal (Pfirrmann grade I),early disc degeneration (Pfirrmann grades II-III), and advanced disc degeneration (Pfirrmann grades IV-V). Regional differences between UTE-T2* and T2* relaxometry and correlation with degeneration were statistically analyzed.

**Results:**

UTE-T2* and T2*value correlated negatively with Pfirrmann grades (*P* < 0.001). In NP, correlations with Pfirrmann grade were high with UTE-T2* values (*r* =  − 0.733; *P* < 0.001) and moderate with T2* values (*r* = -0.654; *P* < 0.001). Diagnostic accuracy of detecting early IVDD was better with UTE-T2* mapping than T2* mapping (*P* < 0.05),with receiver operating characteristic analysis area under the curve of 0.715–0.876.

**Conclusions:**

UTE-T2* relaxometry provides another promising magnetic resonance imaging sequence for quantitatively evaluate lumbar IVDD and was more accurate than T2*mapping in the earlier stage degenerative process.

## Introduction

Low back pain (LBP) is a leading cause of disability worldwide, placing a great burden on the global health care system [1, 2]. Intervertebral disc (IVD) degeneration (IVDD) is a significant contributor to nonspecific LBP, with a lifetime prevalence of over 80% [3, 4].

Early stages of IVDD are mainly in the form of biochemical changes, including proteoglycan (PG) reduction, dehydration, and collagen degeneration. It will lead to a decrease in hydrostatic pressure, resulting in nucleus pulposus (NP) dehydration and loss of the structural and mechanical properties of the IVDs. In advanced stages of IVDD, along with loss of hydration and the subsequent drop in disc pressure, IVD height decreases under load [5–9]. These degenerative changes are accompanied by structural lesions, such as disc herniation, causing LBP, neurogenic claudication, and even cauda equina syndrome. At this stage, treatment strategy is limited to conservative treatment alone or surgical excision [10]. Early detection of alterations in IVDD is important for developing preventative strategies or reestablishing degenerated IVDs, such as gene therapy, stem cell therapy, and growth factor therapy [11–13].

Conventional magnetic resonance imaging (MRI) is widely used for morphologic, qualitative assessment of IVDD in the clinical workup. Lumbar IVDD is commonly scored using the Pfirrmann grading system, which is based on the assessment of structure and loss of the signal intensity on T2-weighted imaging (T2WI). This grading system provides a standardized and reliable assessment of MRI disc morphology, but cannot detect early degeneration of IVDs characterized by a loss of PG quantitatively [14, 15].

Several quantitative MRI techniques to evaluate IVD degeneration objectively have been reported, such as diffusion-weighted imaging, diffusion tensor imaging, glycosaminoglycan chemical exchange saturation transfer, sodium, delayed gadolinium-enhanced MRI, T2/T2*, and T1rho mapping. Previous studies have demonstrated that T2* mapping could be quantitative imaging biomarkers for evaluating the biochemical state of the discs and correlating that with histology, water content, and degeneration [16].

Ultrashort time-to-echo (UTE) imaging as a novel MRI technique has the capacity to catch very short T2* signals (0.008 ~ 0. 50 ms) [17–20], It has been confirmed to be sensitive to changes in the deep tissue matrix and to subtle and even preclinical degeneration [21]. To date, UTE-T2* imaging has been reported to be a reliable tool for quantitative assessment of the biochemical changes of short T2 tissues, including tendon, cartilage, and ligament, etc. [20–25]. However, to the best of our knowledge, studies on UTE-T2* quantitative technique in evaluating IVDD are scarce. We hypothesized that quantitative UTE-T2* mapping is capable of revealing degenerative changes in the discs.

The present study aimed to assess whether lumbar IVDD can be evaluated using UTE-T2* mapping and to compare the potential of UTE-T2*and T2* values in the diagnosis of early IVDD.

## Materials and methods

### Subjects

Ethics approval for this study was provided by the ethics commission of the Fudan University Affiliated Zhongshan Hospital Xiamen Branch. Written informed consent was obtained from all subjects. The inclusion criteria were patients with single or recurrent episodes of nonspecific LBP in the last 6 months and age ≥ 18 years. Exclusion criteria were contraindications for MRI and patients with other spine diseases, such as spinal infection, tumor, tuberculosis, and serious scoliosis.

### MRI protocols

All data were acquired on a 3.0 T MRI scanner (Discovery™ MR750w, GE Healthcare, Milwaukee, WI) equipped with an 8-channel spine-array coil covering the IVDs L1/L2 to L5/S1. All participants underwent MRI examinations, including sagittal T2WI, UTE-T2*, and T2* mapping. T2WI images were used for Pfirrmann grading. Detailed acquisition parameters were assigned to T2WI, UTE-T2* mapping, and T2* mapping.

For T2WI, repetition time (TR)/echo time (TE) = 2500/120 ms, slice thickness/gap = 5/0.5 mm, field of view (FOV) = 300 × 300 mm, matrix = 256 × 192, and bandwidth = 41.7 kHz. For UTE-T2* mapping, TR = 112.4 ms, TE = 0.032/3.8/8.8/13.2 ms, slice thickness/gap = 3/0.5 mm, flip angle = 15, FOV = 300 × 300 mm, matrix = 300 × 256, and bandwidth = 62.5 kHz. Maps of the T2* was calculated using R2*(R2* = 1/T2*) [26, 27].For R2* mapping, TR = 12.5 ms, TE = 1.40 /2.29 /3.18/4.07/ 4.96/5.85/ 6.74 / 7.63 / 8.52 ms, slice thickness/gap = 5/0 mm, flip angle = 5, FOV = 300 × 300 mm, matrix = 256 × 192, and bandwidth = 111.1 kHz.

### Image analysis

**Fig. 1:**
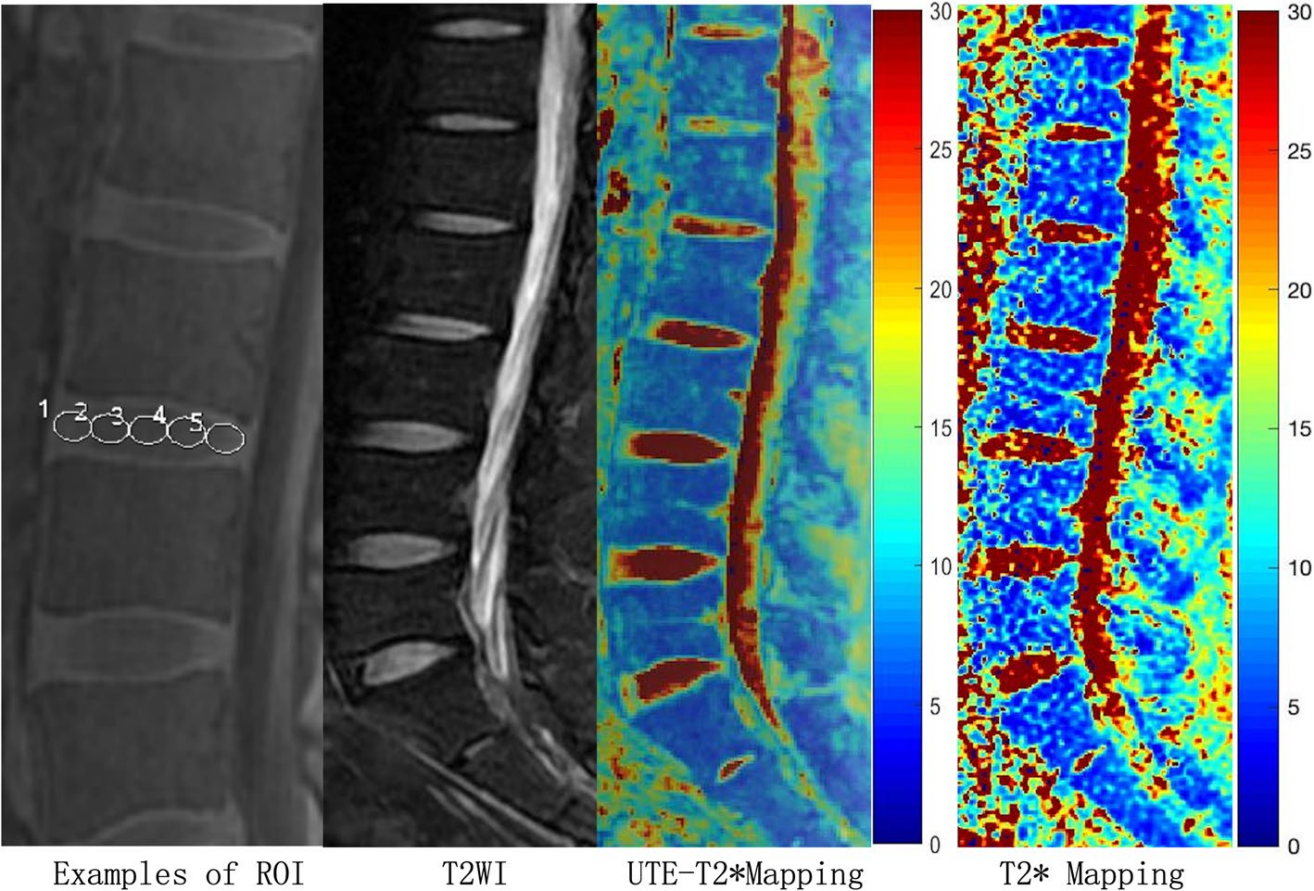
MR images of the lumbar spine of a 39-year-old woman. Every lumbar IVD was cut into 5 uniform parts in each UTE-T2* and T2* mapping. An ROI of 1 represented AAF, ROI 2–4 represented NP, and ROI 5 represented PAF. IVD, intervertebral disc; NP, nucleus pulposus; PAF, posterior annulus fibrosus; ROI, region of interest; UTE, ultrashort time-to-echo

All lumbar IVDs were evaluated by 2 musculoskeletal radiologists each with more than six years of experience using T2WI and assigned a Pfirrmann grade (**Table ****1**) [28]. Disc degenerations also were divided into three categories [11]: normal (Pfirrmann grade I), early disc degeneration (Pfirrmann grade II-III), and advanced disc degeneration (Pfirrmann grade IV-V). UTE-T2* values were calculated by mono-exponential fitting using custom code in MATLAB (MathWorks, Natick, MA). Five circular regions of interest (ROIs) of equal size were manually drawn on the midline slice of sagittal UTE-T2* and T2* mappings from anterior to posterior (**Fig. ****1**), including the anterior annulus fibrosus (AAF; ROI 1), nucleus pulposus (NP; ROI 2–4), and posterior annulus fibrosus (PAF; ROI 5) [29].

**Table 1:** Pfirrmann grades of disc degeneration

Grade	Structure	Distinction of nucleus and annulus	Signal intensity	Height of intervertebral disc
I	Homogeneous, bright white	Clear	Hyperintense, isointense to cerebrospinal fluid	Normal
II	Inhomogeneous with or without horizontal bands	Clear	Hyperintense, isointense to cerebrospinal fluid	Normal
III	Inhomogeneous, gray	Unclear	Intermediate	Normal to slightly decreased
IV	Inhomogeneous, gray to black	Lost	Intermediate to hypointense	Normal to moderately decreased
V	Inhomogeneous, black	Lost	Hypointense	Collapsed disc space

### Statistical analysis

Statistical analysis was conducted using SPSS 22.0 software (IBM, Armonk, NY) and Medcalc 20.022 (Mariakerke, Belgium). The Kruskal–Wallis test was performed to determine differences among the 5-level Pfirrmann grades. The differences between the two methods were expressed using the ± 95% confidence intervals (CIs) from the Bland–Altman analysis. Correlations of quantitative values with Pfirrmann grades were analyzed using Spearman's rank correlation. Receiver operating characteristic (ROC) analysis was performed and area under the curve (AUC), sensitivity, specificities, positive likelihood ratio (+ LR), and negative likelihood ratio (− LR) were obtained to assess the diagnostic efficacy of each quantitative parameter for differentiating normal IVDs from early disc degeneration and to differentiate early disc degeneration from advanced disc degeneration. AUCs were compared using the DeLong method [30]. A *P* value less than 0.05 was considered statistically significant.

## Results

### Clinical characteristics

Seventy-six subjects aged 54.4 ± 15.7 years (range, 19–85 years) were recruited into this study. They included 29 males (aged 51.8 ± 17.2 years; range, 19–82 years) and 47 females (aged 54.5 ± 14.9 years; range, 27–85 years). Five intervertebral discs (L1/L2–L5/S1) per subject were examined, of which 14 discs were excluded due to previous vertebral fusion operation (*n* = 2), collapsed disc space (Pfirrmann grade V) that made it impossible to measure the quantitative values (*n* = 9), and image quality problems (*n* = 3). A total of 366 IVDs were included in this study.

**Fig. 2:**
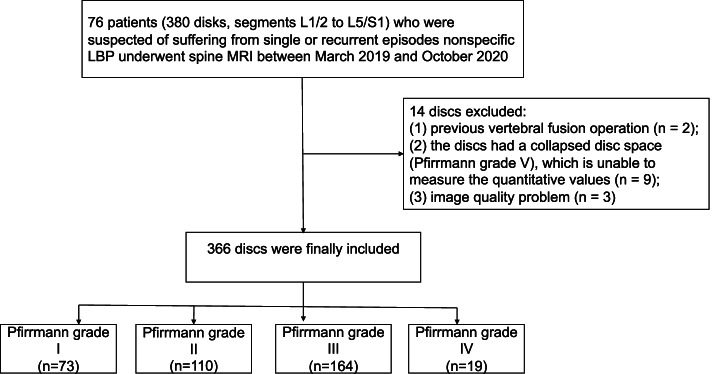
Flow diagram of the study enrolment discs

Using the Pfirrmann grading system, 73 discs were categorized as grade I; 110 discs, as grade II; 164 discs, as grade III; and 19 discs, as grade IV. The flowchart for the enrollment of the study population is presented in **Fig. ****2**. The distribution of the UTE-T2*and T2* values with respect to Pfirrmann grades is provided in **Table ****2**.

**Table 2:** UTE-T2* and T2* values of AAF, NP, and PAF in each Pfirrmann grades

Number		Pfirrmann grade				*P* value
		I	II	III	IV	
		73	110	164	19	
AAF	UTE-T2*	23.5 ± 5.6	19.9 ± 4.7	19.2 (5.8)	15.9 ± 3.4	< 0.001
	T2*	21.2 (8.0)	20.0 (7.4)	16.9 (5.9)	16.2 (11.1)	< 0.001
NP	UTE-T2*	46.6 ± 10.1	35.9 (9.9)	27.8 ± 5.9	20.0 ± 5.3	< 0.001
	T2*	48.3 (19.9)	43.3 (17.8)	29.7 (9.9)	22.8 ± 5.8	< 0.001
PAF	UTE-T2*	24.6 ± 3.6	20.6 ± 5.7	18.7 (6.5)	11.9 (8.3)	< 0.001
	T2*	19.7 ± 4.8	17.3 (7.0)	15.2 (5.4)	11.8 (6.3)	< 0.001

*AAF* Anterior annulus fibrosus, *NP* Nucleus pulposus, *PAF* Posterior annulus fibrosus, *UTE* Ultrashort time-to-echo

distribution data is expressed as mean ± standard deviation

skewed data is expressed as median, quartile spacing

UTE-T2* and T2*value is given in ms

### Correlation of UTE-T2*and T2* values with Pfirrmann grades

**Fig. 3:**
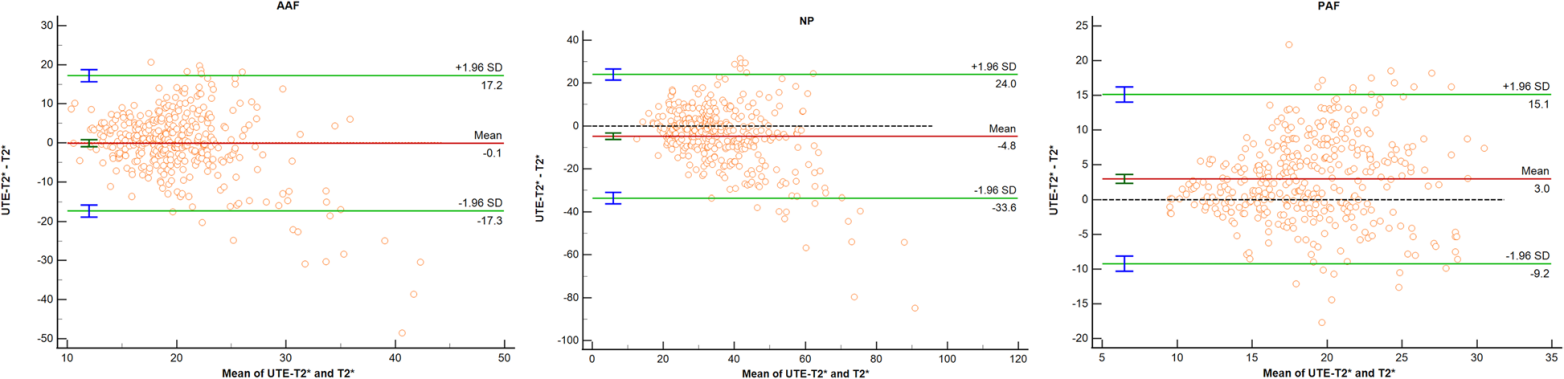
Bland–Altman plots comparing both UTE-T2* and T2* values of AAF, NP, PAF. Bias (solid line) and limits of agreement (dashed line) are shown for each variable. The mean score is plotted on the x-axis, while the difference between the two methods is plotted on the y-axis (mean difference ± 1.96 SD). AAF, anterior annulus fibrosus; NP, nucleus pulposus; PAF, posterior annulus fibrosus

**Fig. 4:**
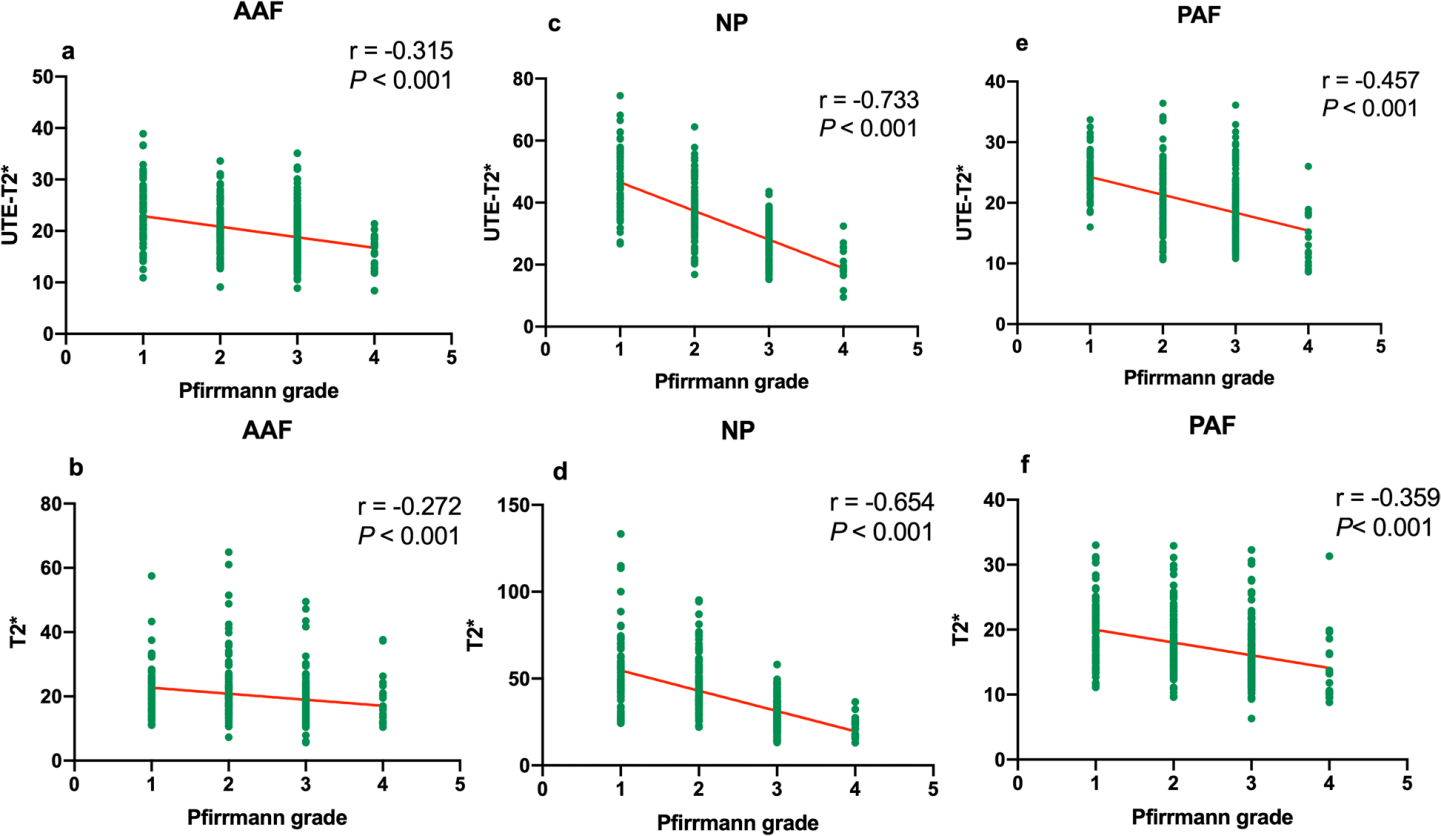
Scatter plots of the values in AAF, NP, and PAF according to the Pfirrmann grades. a, c, and e are respectively UTE-T2* relaxation time of AAF, NP, and PAF correlated with disc degeneration grading; b, d, and f are respectively T2* value of AAF, NP, and PAF correlated with disc degeneration grading

The Kruskal–Wallis test demonstrated that all quantitative values for all segments were significantly different among different Pfirrmann grades (**Table ****2**). Bland–Altman plots are shown in **Fig. ****3**. There was no significant bias between UTE-T2*and T2* values in NP and PAF(*P* > 0.05).UTE-T2* values showed high correlations with Pfirrmann grade in NP (*r* =  − 0.733; *P* < 0.001). Moderate correlations with Pfirrmann grade were observed in T2* value of NP (*r* = -0.654; *P* < 0.001). Comparing the Spearman correlation coefficient, the highest correlation value was seen in NP and the lowest was seen in AAF. Among those, the UTE-T2* value of NP showed the highest correlation values with Pfirrmann grades. Results of Spearman's correlation analysis are summarized in **Fig. ****4**.

### Post hoc multiple comparisons among each Pfirrmann grades

**Fig. 5:**
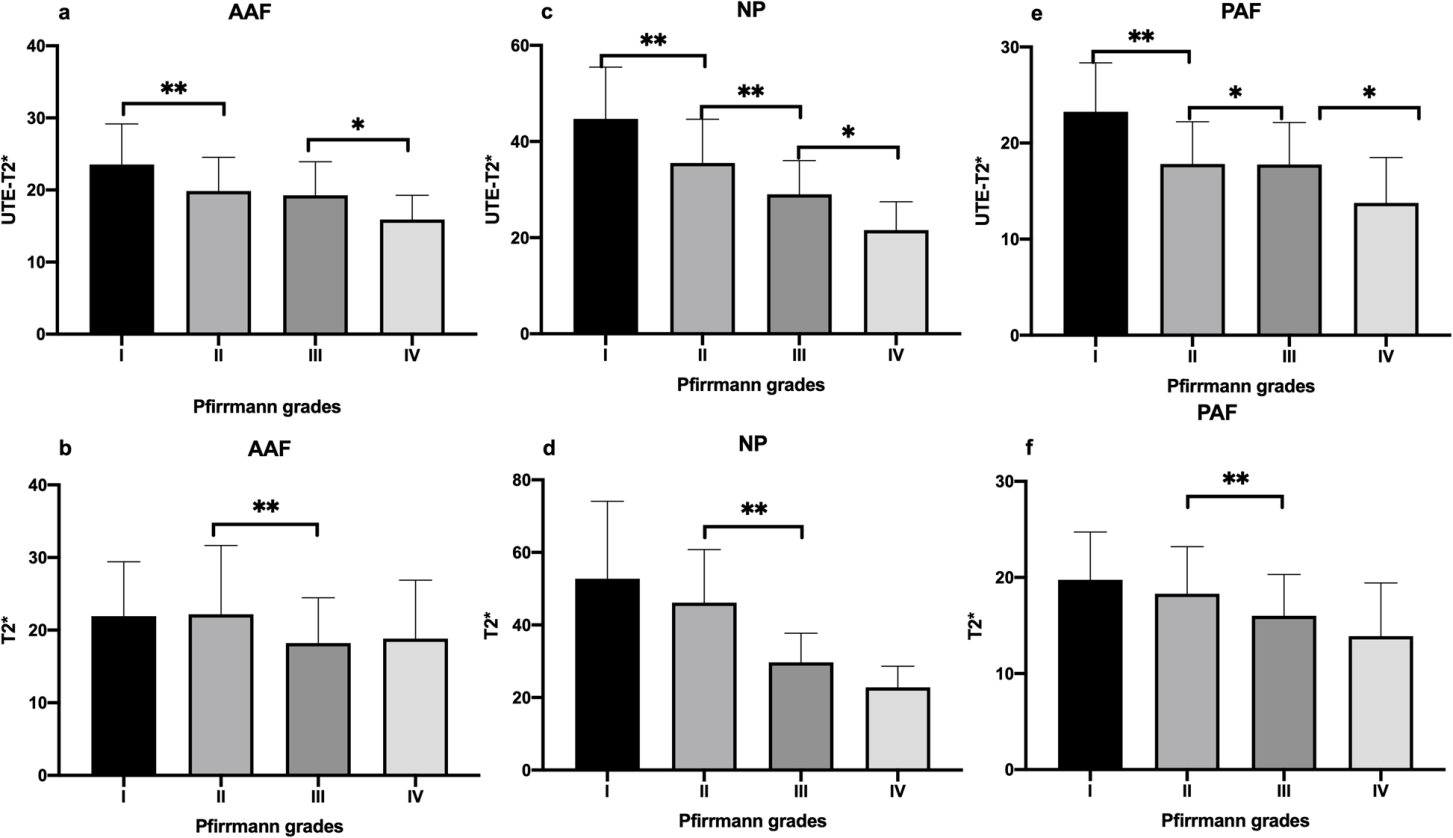
Post hoc multiple comparisons among 4 Pfirrmann grades. There were significant differences in UTE-T2* values of NP and PAF between each Pfirrmann grades. T2* values were found to be significantly different between Pfirrmann grades II and III in AAF, NP, and PAF. *P values of < 0.05 and **P values of < 0.001 were considered statistically significant. NP, nucleus pulposus; PAF, posterior annulus fibrosus; UTE, ultrashort time-to-echo

There were significant differences in UTE-T2* values of NP and PAF between each Pfirrmann grade. T2* values were found to be significantly different between Pfirrmann grade II and grade III in AAF, NP, and PAF (**Fig. ****5**).

### Diagnostic performance of UTE-T2* and T2* values in distinguishing each degeneration groups

**Fig. 6:**
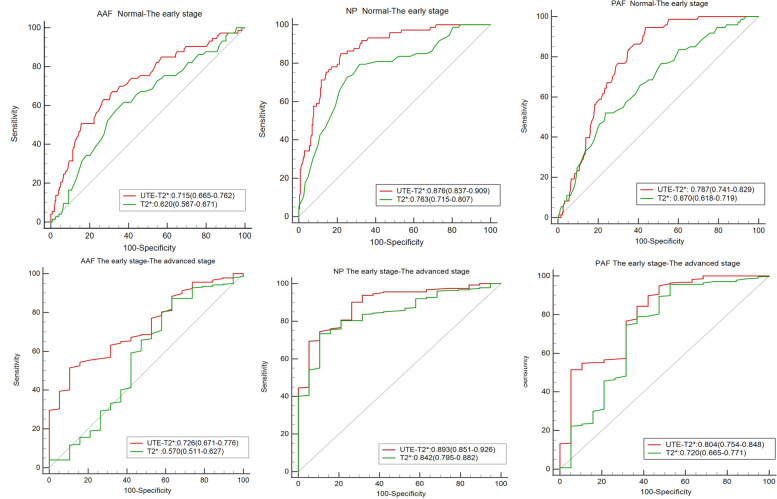
Receiver operating characteristic (ROC) analysis. Graphs show ROC curves to distinguish each degeneration groups. Numbers are areas under the curves with 95% confidence intervals in parentheses

ROC curves of UTE-T2* and T2* values for distinguishing each degeneration groups are plotted in **Fig. ****6** The corresponding diagnostic test characteristics are provided in **Table ****3**. The AUC values of UTE-T2* mapping in AAF, NP, and PAF were 0.715,0.876,0.787, respectively, for identification of the early disc degeneration, and 0.726,0.893,0.804, respectively, for identification of the advanced disc degeneration. The AUC values of T2* mapping in AAF, NP, and PAF were 0.620, 0.763, 0.670, respectively, for identification of the early disc degeneration, and 0.570, 0.842, 0.720, respectively, for identification of the advanced disc degeneration.

**Table 3:** Diagnostic performance of UTE-T2* and T2* values in distinguishing degeneration groups

3a. Normal-The early stage								
		AUC (95% CI)	Sensitivity (%)	Specificity (%)	+ LR		-LR	Cutoff
AAF	UTE-T2*	0.715 (0.665–0.762)	73.0	63.0	1.97		0.43	≤ 21.8
	T2*	0.620 (0.567–0.671)	66.1	58.9	1.61		0.58	≤ 20.1
NP	UTE-T2*	0.876 (0.837–0.909)	78.1	84.9	5.18		0.26	≤ 36.7
	T2*	0.763 (0.715–0.807)	68.3	79.5	3.32		0.40	≤ 39.2
PAF	UTE-T2*	0.787 (0.741–0.829)	56.6	94.5	10.32		0.46	≤ 19.6
	T2*	0.670 (0.618–0.719)	76.3	52.1	1.59		0.46	≤ 19.4
3b. The early stage – The advanced stage								
		AUC (95% CI)	Sensitivity (%)	Specificity (%)	+ LR	-LR		Cutoff
AAF	UTE-T2*	0.726 (0.671–0.776)	89.5	51.5	1.84	0.20		≤ 19.1
	T2*	0.570 (0.511–0.627)#	36.8	87.2	2.88	0.72		≤ 13.6
NP	UTE-T2*	0.893 (0.851–0.926)	94.7	69.3	3.09	0.08		≤ 27.0
	T2*	0.842 (0.795–0.882)	89.5	73.4	3.36	0.14		≤ 27.4
PAF	UTE-T2*	0.804 (0.754–0.848)	57.9	89.8	5.67	0.47		≤ 13.0
	T2*	0.720 (0.665–0.771)	47.4	95.6	10.82	0.55		≤ 10.6

^#^*P* values of > 0.05 were considered without statistical significance

UTE-T2* and T2*value is given in ms

For pairwise comparisons of ROC curves, UTE-T2* values in NP and PAF were better in identifying early degeneration IVDs than those in T2*. There were no significant differences among UTE-T2* and T2* mapping in AAF. Comparing between different segments, diagnostic performance of NP was the highest in predicting the early degeneration IVDs, AAF and PAF performed similarly. For differentiating early and advanced disc degeneration, the UTE-T2* value of PAF was better than that of T2* and there were no significant differences among UTE-T2* and T2* mapping in AAF and NP. Comparing between different segments, diagnostic performance of NP was better than AAF, AAF and PAF performed similarly in predicting the advanced disc degeneration.

Overall, the diagnostic efficacy of UTE T2* mapping was better than that of T2* mapping for evaluating IVDD, especially in NP. The results of this study demonstrated that the UTE-T2* value in NP showed high correlations with Pfirrmann grade (*r* =  − 0.733; *P* < 0.001) and that AUCs for the assessment of the early disc degeneration (AUC 0.876) were significantly higher than those for T2*(AUC 0.763).

## Discussion

This study is the first to investigate and compare the diagnostic efficacies of UTE-T2* and T2* mapping in detecting IVDD in humans. The results may help to confirm the feasibility and specificity of UTE-T2* as an objective and quantitative tool to identify early degenerative changes of the disc and show promise for clinicians to modify the diagnostics and therapeutic management strategies more accurately.

Conventional MRI, such as the Pfirrmann scale with T2WI, was limited in detecting ultrastructural alterations of early IVDD. Early stages of disc degeneration include biochemical changes, such as a loss or reduction of PG content, which can ultimately lead to dehydration. T2 relaxation reflected the integrated environment of the IVD, including water, protein, collagen, and other solutes [31], and was sensitive to water content and the composition of the collagen network structure. Researchers have reported that T2* relaxation time showed a good correlation with PG and collagen contents in 18 humans cadaveric IVDs [32]. Our findings confirmed an increase in the quantitative T2* values between the AAF and NP and a decrease between the NP and the PAF. In line with an earlier report [33, 34],T2 and T2* mapping provided roughly similar results. The inverse correlation of the T2 relaxation time in the disc with Pfirrmann grade has been reported by Welsch et al. [35] and Noebauer et al. [36], the early study reported a low-to-moderate correlation between Pfirrmann grades and T2 relaxation times, which were consistent with the Spearman's correlation coefficient between Pfirrmann grades and T2*value in our results. Both T2 and T2* mapping differ in the biochemical sensitivity of disc tissue, with T2 mapping being sensitive to tissue hydration, while T2* mapping being more sensitive to changes in tissue integrity [35].T2* mapping provide more valuable biochemical information on the IVDs ultrastructure, together with three-dimensional acquisition capability and higher spatial resolution in a short scan time [37].

UTE-T2* mapping was acquired using different echo times in the short (1–10 ms) and ultrashort echo time range [19, 22]. Because UTE-T2* mapping can catch the short T2* relaxations from tissues, it is more sensitive to biochemical collagen matrix changes compared with conventional MRI techniques, based on histologic standards [19].

Multiple large general-population–based studies have proved that UTE-T2* mapping can detect cartilage subsurface matrix changes, which can be indicative of reduced cartilage health from injury or early degeneration noninvasively [20, 23, 25]. Similar to the results reported by Detiger et al. [16], we observed a trend of decreasing UTE-T2* value with increasing degree of degeneration. The previous study revealed a significant correlation between T2* relaxation time and glycosaminoglycans (GAG) content in the nucleus pulposus, as well as histologic scoring with varying grades of degeneration [16]. During the aging process, the quantity and quality of PG and collagen contents diminish, along with a decrease in short T2* signal accordingly [21, 25, 34, 38]. T2* relaxometry appeared to be sensitive to water and PG contents. This may be the initial step in the degenerative cycle [25, 34, 38], which could be the underlying reasons for the decreased UTE-T2* and T2* value.

In NP and PAF, UTE-T2* mapping showed significantly higher diagnostic accuracy in differentiating early disc degeneration from normal than did T2*. Theoretically, both UTE-T2* and T2* mapping measured the T2* value of the tissue. UTE MRI technique mitigates the rapid signal loss from short T2* by reducing the TE to the scale of 0–200 microseconds to sample the free induction decay as early as possible. With considerably shorter TEs (0.032 ms in this study) than T2*, UTE-T2*mapping allows signals from very short T2 components to be detected [23]. Thus, UTE-T2*mapping is less sensitive to the magic angle effect and more sensitive to water protons and their local environment, making it a satisfactory method for evaluating disc generation. Because T2* relaxation time has been reported to reflect both the water content and PG content reduction [16], it is not hard to understand why the UTE-T2* value has better diagnostic accuracy than T2* for differentiating early disc degeneration.

Previous studies have reported that T2 relaxation time of Pfirrmann grades IV is significantly shorter than Pfirrmann grades III, and no significant differences were found between Pfirrmann grades IV and V, both of which show extremely low signal intensity [39]. The results of our study showed that compared to T2* values, UTE-T2* values conveyed significantly higher diagnostic performance in distinguishing the early disc degeneration from the advanced in PAF. Takashima et al. [40] reported that short T2* relaxation times with UTE are promising for assessing progressive IVD degeneration with poor water content, such as fibrosis change of IVDD with short T2 relaxation time. Our results are consistent with those findings quantitatively. Takashima et al. did not further discuss the quantitative evaluation of the early disc degeneration because their study population did not include grade I IVDs. Our results would appear to complement and refine their research.

A previous report on healthy ovine IVDs demonstrated that the T2 values show regional variation in discs and reported that high T2 values were observed in NP and low T2 values in the AAF and PAF when histologically evaluated [41]. Disc degeneration is believed to originates in NP with depletion of GAG, followed by a reduction in water content [5, 42]. Similar to previous reports, our study showed that correlations with Pfirrmann grade and UTE-T2* and T2* values were highest in NP and lowest in AAF. Our results also showed that NP had the highest diagnostic accuracy in predicting the early degeneration IVDs, while AFP and PFP were similar in predicting the early degeneration IVDs. These results suggest that the destruction of hydrophilic GAGs within NP was the main cause of the accumulation of cleaved extracellular matrix fragments with disc aging [16].

There were some limitations in this study. First, our study had no detailed histologic confirmation associated with IVDD changes. This is hard to achieve in humans. In addition, the relationship between T2* values and biochemical changes in IVDD has been previously established in human cadaveric lumbar discs [32]. Second, we were unable to compare the related clinical symptoms with the degree of degeneration in MRI quantitative parameters. Future research is warranted to explore these ideas. Third, no patient in this study showed grade V IVDs because grade V IVDs tend to have a collapsed disc space or a vacuum phenomenon, which is unable to measure the quantitative values due to susceptibility artifacts. If the complete grade V IVDs data set were available, quantitative evaluation of advanced IVD degeneration could be closer to reality. However, as our principal purpose was to detect early disc degeneration, the impact of incomplete grade V IVDs dataset on our results is within acceptable limits.

## Conclusions

We demonstrated that UTE-T2*mapping was more accurate than T2* mapping in quantitatively diagnosing early intervertebral disc degeneration. In particular, UTE-T2* mapping allowed for precisely distinguishing disc degeneration, potentially providing a promising imaging biomarker with potential applications in intervertebral disc degeneration for the emerging cell-based therapies.

## Data Availability

The datasets generated and/or analysed during the current study are available from the corresponding author on reasonable request.

## Abbreviations

LBP: Low back pain
IVD: Intervertebral disc
IVDD: Intervertebral disc degeneration
PG: Proteoglycan
NP: Nucleus pulposus
T2WI: T2-weighted imaging
UTE: Ultrashort time-to-echo
TR: Repetition time
TE: Echo time
FOV: Field of view
PAF: Posterior annulus fibrosus
AAF: Anterior annulus fibrosus
ROI: Region of interest
ROC: Receiver operating characteristic
